# A systematic review of comparisons between protocols or registrations and full reports in primary biomedical research

**DOI:** 10.1186/s12874-017-0465-7

**Published:** 2018-01-11

**Authors:** Guowei Li, Luciana P. F. Abbade, Ikunna Nwosu, Yanling Jin, Alvin Leenus, Muhammad Maaz, Mei Wang, Meha Bhatt, Laura Zielinski, Nitika Sanger, Bianca Bantoto, Candice Luo, Ieta Shams, Hamnah Shahid, Yaping Chang, Guangwen Sun, Lawrence Mbuagbaw, Zainab Samaan, Mitchell A. H. Levine, Jonathan D. Adachi, Lehana Thabane

**Affiliations:** 1 Department of Health Research Methods, Evidence, and Impact, McMaster University, St. Joseph’s Healthcare, Hamilton, 501-25 Charlton Avenue East, Hamilton, ON L8N 1Y2 Canada; 2 St. Joseph’s Healthcare Hamilton, McMaster University, Hamilton, ON Canada; 3 Centre for Evaluation of Medicines, Programs for Assessment of Technology in Health (PATH) Research Institute, McMaster University, Hamilton, ON Canada; 4 Department of Dermatology and Radiotherapy, Botucatu Medical School, Universidade Estadual Paulista, UNESP, São Paulo, Brazil; 5 Faculty of Health Sciences, McMaster University, Hamilton, ON Canada; 6 McMaster Integrative Neuroscience Discovery and Study, McMaster University, Hamilton, ON Canada; 7 Medical Sciences, McMaster University, Hamilton, ON Canada; 8 Integrated Sciences, McMaster University, Hamilton, ON Canada; 9 Psychology, Neuroscience and Behaviour, McMaster University, Hamilton, ON Canada; 10 Arts and Science, McMaster University, Hamilton, ON Canada; 11 Department of Medicine, McMaster University, Hamilton, Canada; 12 Department of Health Research Methods, Evidence, and Impact, McMaster University, Father Sean O’Sullivan Research Centre, St. Joseph’s Healthcare Hamilton, 3rd Floor Martha, Room H325, 50 Charlton Avenue E, Hamilton, ON L8N 4A6 Canada

**Keywords:** Protocol, Registration, Selective reporting, Discrepancy, Inconsistency, Biased reporting, Incomplete reporting

## Abstract

**Background:**

Prospective study protocols and registrations can play a significant role in reducing incomplete or selective reporting of primary biomedical research, because they are pre-specified blueprints which are available for the evaluation of, and comparison with, full reports. However, inconsistencies between protocols or registrations and full reports have been frequently documented. In this systematic review, which forms part of our series on the state of reporting of primary biomedical, we aimed to survey the existing evidence of inconsistencies between protocols or registrations (i.e., *what was planned to be done* and/or *what was actually done*) and full reports (i.e., *what was reported in the literature*); this was based on findings from systematic reviews and surveys in the literature.

**Methods:**

Electronic databases, including CINAHL, MEDLINE, Web of Science, and EMBASE, were searched to identify eligible surveys and systematic reviews. Our primary outcome was the level of inconsistency (expressed as a percentage, with higher percentages indicating greater inconsistency) between protocols or registration and full reports. We summarized the findings from the included systematic reviews and surveys qualitatively.

**Results:**

There were 37 studies (33 surveys and 4 systematic reviews) included in our analyses. Most studies (*n* = 36) compared protocols or registrations with full reports in clinical trials, while a single survey focused on primary studies of clinical trials and observational research. High inconsistency levels were found in outcome reporting (ranging from 14% to 100%), subgroup reporting (from 12% to 100%), statistical analyses (from 9% to 47%), and other measure comparisons. Some factors, such as outcomes with significant results, sponsorship, type of outcome and disease speciality were reported to be significantly related to inconsistent reporting.

**Conclusions:**

We found that inconsistent reporting between protocols or registrations and full reports of primary biomedical research is frequent, prevalent and suboptimal. We also identified methodological issues such as the need for consensus on measuring inconsistency across sources for trial reports, and more studies evaluating transparency and reproducibility in reporting all aspects of study design and analysis. A joint effort involving authors, journals, sponsors, regulators and research ethics committees is required to solve this problem.

## Background

Incomplete or selective reporting in publications is a serious threat to the validity of findings from primary biomedical research, because inadequate reporting may be subject to bias, and it subsequently impairs evidence-based decision-making [1, 2]. Prospective study protocols and registrations can play a significant role in reducing incomplete or selective reporting, because they are pre-specified blueprints which are available for the evaluation of, and comparison with, full reports [3, 4]. Therefore, for instance, in 2004 the International Committee of Medical Journal Editors (ICMJE) stated that all trials must be included in a trial registry before participant enrollment as a compulsory condition of publication [5], because registry records may include information on either what was planned or what was done during a study. Moreover, one recent study reported that primary outcomes were more consistently reported when a trial had been prospectively registered [6]. With wide acceptance of trial registration, many journals started establishing editorial policies to publish protocols. However, inconsistency was found to be strikingly frequent after comparing protocols or registrations with full reports regarding outcome reporting, subgroup selection, sample size, statistical analysis, among others [7–11]. In this systematic review, which forms part of our series on the state of reporting of primary biomedical research, our objectives were to map the existing evidence of inconsistency between protocols or registrations (i.e., *what was planned to be done* and/or *what was actually done*) and full reports (i.e., *what was reported in the literature*), and to provide recommendations to mitigate such inconsistent reporting, based on findings from systematic reviews and surveys in the literature [12].

## Methods

We followed the guidance from the Joanna Briggs Institute [13] and/or the PRISMA (Preferred Reporting Items for Systematic Reviews and Meta-Analyses) [14] to conduct and report our review. Details on the methods have been published in our protocol [12].

### Eligibility and search strategy

In brief, in this systematic review, we included systematic reviews or surveys that focused on inconsistent reporting when comparing protocols or registration with full reports. A study protocol is defined as the original research plan with comprehensive description of study participants or subjects, outcomes, objective(s), design, methodology, statistical consideration and other related information that cannot be influenced by the subsequent study results. In this review, we defined full reports as the publications that included findings of any of the study key elements, including participants or subjects, interventions or exposures, controls, outcomes, time frames, study designs, analyses, result interpretations and conclusions, and other study-related information, and that had been published after completion of the studies. Therefore full reports may include full-length articles, research letters, or other published reports without peer review. An eligible systematic review was defined as a study that assessed the comparisons between protocols or registrations and full reports, and that had predefined objectives, specified eligibility criteria, at least one database searched, data extraction and analyses, and at least one study included. All the surveys that included primary studies and that compared protocols or registrations and full reports were eligible for inclusion.

Exclusion criteria were: 1) the study was not a systematic review or survey, 2) the study objective did not include comparison between protocols or registration with full reports, 3) the study could not provide data on such comparisons, 4) the study did not focus on primary biomedical studies, or 5) the study was in duplicate. The search process was completed by one reviewer (GL) with the help of an experienced librarian. It was limited to several databases (CINAHL, EMBASE, Web of Science, and MEDLINE) from 1996 to September 30th 2016, restricted to studies in English. Two reviewers (YJ and IN) independently screened the records retrieved from the search. Reference lists from the included studies were also searched by hand in duplicate by the two reviewers (YJ and IN), to avoid the omission of potentially eligible systematic reviews and surveys. The kappa statistic was used to assess the agreement level between the two reviewers [15].

### Outcome and data collection

Our primary outcome was the percentage of primary studies in the included systematic reviews and surveys for which an inconsistency was observed between the protocol or registration and the full report, with higher percentages indicating greater inconsistency [12]. Inconsistencies were recorded between protocols or registrations and full reports with respect to study participants or subjects, interventions or exposures, controls, outcomes, time frames, study designs, analyses, result interpretations and conclusions, and other study-related information. A secondary outcome was the factors reported to be significantly associated with the inconsistency between protocols or registration and full reports.

Two independent reviewers (IN and LA) extracted the data from the included studies. Data collected included the general characteristics of the systematic reviews or surveys (author, year of publication, journal, study area, data sources, search frame, numbers and study designs of included primary studies for each systematic review or survey, measure of comparison, country and sample size of primary studies, and funding information), key findings of inconsistent reporting, authors’ conclusions, and the factors reported to be significantly related to inconsistent reporting. The terminologies and their frequency used in the included systematic reviews and surveys to describe the reporting problem were also collected.

### Quality assessment and data analyses

Study quality was assessed for the included systematic reviews using the AMSTAR (a measurement tool to assess systematic reviews) [16]; no comparable assessment tool was available for surveys. We excluded two items of the AMSTAR (item 9 “Were the methods used to combine the findings of studies appropriate?” and 10 “Was the likelihood of publication bias assessed?”) because they were not relevant to the included systematic reviews.

Inconsistency was analysed descriptively using medians and interquartile ranges (IQRs). Frequencies of the terminologies that were used to describe the inconsistent reporting problem and that were extracted from the included systematic reviews and surveys were calculated and shown by using word clouds. The word clouds were generated using the online program Wordle (www.wordle.net) with the input of the terminologies and their frequencies. The relative size of the terms in the word clouds corresponded to the frequency of their use. We summarized findings from the included systematic reviews and surveys qualitatively. No pooled analyses were performed in this review.

## Results

**Fig. 1:**
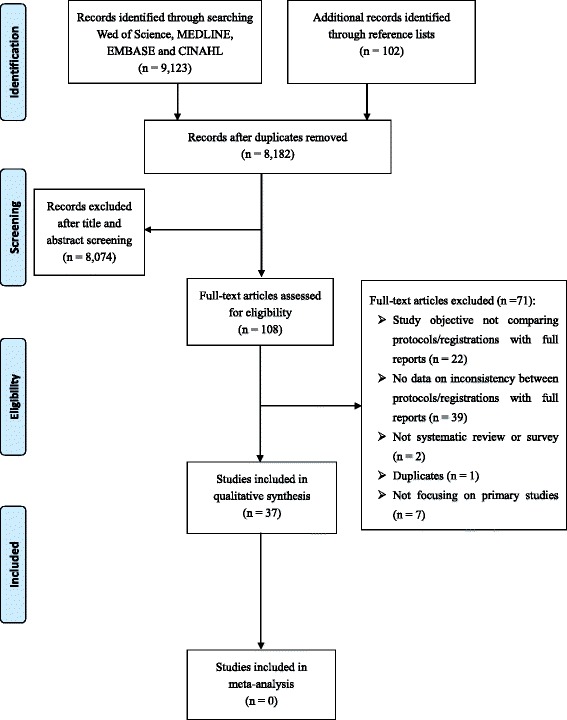
Study flow diagram showing the study selection process

A total of 9123 records were retrieved. After removing duplicates, 8080 records were screened through their titles and abstracts. There were 108 studies accessed for full-text article evaluation (kappa = 0.81, 95% confidence interval: 0.75–0.86). We included 37 studies (33 surveys and 4 systematic reviews) for analysis [7–11, 17–48]. Fig. 1 shows the study inclusion process.

Table 1 displays the characteristics of the included studies that were published between 2002 and 2016. Approximately half studies (*n* = 13) focused on composite areas in biomedicine, five studies on surgery or orthopaedics, four on oncology, and four on pharmacotherapeutic studies. There were 15 studies that had collected data from the registry entries, with the most commonly-used registries being ClinicalTrials.gov (*n* = 13), ISRCTN (the International Standard Randomised Controlled Trial Number Registry; *n* = 8), and WHO ICTRP (World Health Organization International Clinical Trials Registry Platform; *n* = 7). Eight studies collected protocol data from grant or ethics applications, two from FDA (Food and Drug Administration) reviews, two from internal company documents, one from a journal’s website, and nine from other sources, respectively. Regarding the data sources for full reports, most studies (*n* = 35) searched databases and/or journal websites to access full reports, while one study collected full reports by searching databases and contacting investigators [17] and another study by contacting the lead investigators only [20]. Most systematic reviews or surveys (*n* = 36) compared protocols or registrations with full reports in clinical trials; there was only one included survey that investigated inconsistent reporting in both clinical trials and observational research [18]. Measures of comparison between protocols or registrations and full reports included outcome reporting, subgroup reporting, statistical analyses, sample size, participant inclusion criteria, randomization, and funding, among others (Table 1). Most primary studies were conducted in North America and Europe. Among the included systematic reviews and surveys that reported information on sample sizes for the primary studies, the median sample sizes in the primary studies ranged from 16 to 463. There were 15 studies that had received academic funding for their conduct of a systematic review or survey, and 2 studies that had received litigation-related consultant fees.

**Table 1:** Study characteristics including study areas, data sources, search frames, numbers and designs of included primary research, measures of comparison, and other related information for the included 33 surveys and 4 systematic reviews

First author, publication year, journal	Study area	Data sources for protocols /registrations	Data sources for full reports	Study search frame^b^	Numbers of protocols (or registrations)/full reports	Study design of primary studies	Measures of comparison between protocols /registrations and full reports	Study country of primary studies	Study sample size of full reports in primary studies	Funding for the systematic review or survey
*Survey (n = 33)*										
Al-Marzouki, 2008, The Lancet [17]	Composite areas	Protocols published on The Lancet’s website	Contacting investigators and database searching	Protocols: As of June, 2007	64/37	RCTs	Selective outcome reporting and subgroup analyses	Not reported	Not reported	Not reported
Chan, 2004, Canadian Medical Association or its licensors [19]	Composite areas	Protocols approved by Canadian Institutes of Health Research (CIHR) or Medical Research Council of Canada (MRC)	PubMed, EMBASE and the Cochrane Controlled Trials Register	Protocols: 1990–1998; Full reports: up to 2003	48/68	RCTs	Outcome reporting	Canada	Median sample size: 299	Not reported
Chan, 2004, Journal of the American Medical Association [7]	Composite areas	Protocols approved by Scientific-Ethical Committees for Copenhagen and Frederiksberg’ Denmark	Contacting trialists and by searching MEDLINE, EMBASE, and the Cochrane Controlled Trials Register	Protocols: 1994–1995; Full reports: up to May 2003	102/122	RCTS	Outcome reporting	Denmark	Median sample size: 151 for parallel RCTs; 16 for crossover RCTs	Academic funding
Chan, 2008, British Medical Journal [8]	Composite areas	Protocols approved by Scientific-Ethical Committees for Copenhagen and Frederiksberg, Denmark	PubMed, Embase, and the Cochrane Controlled Trials Register	Protocols: from January 1994 to December 1995	70/70	RCTs	Sample size calculations and data analyses	Denmark	Median sample size: 66 for per study arm	No funding received
Hartung, 2014, Annals of Internal Medicine [22]	Composite area	ClinicalTrials.gov	PubMed or the National Library of Medicine	Protocols: if trials completed before January 2009, or trials starting before July 2008 if primary completion date was unknown	110/110	RCTs	Outcome reporting, study sample and results presentations	Not reported	Median sample size: 352	Academic funding
Kasenda, 2014, British Medical Journal [9]	Composite areas	Protocols approved by six research ethics committees in Switzerland (Basel, Lucerne, Zurich, and Lausanne), Germany (Freiburg), and Canada (Hamilton)	Publications of trials from corresponding protocols	Protocols: approved between 2000 and 2003	894/515	RCTs	Subgroup analyses	Switzerland, Germany, and Canada.	Median sample size: 260	Academic funding
Redmond, 2013, Journal of Clinical Epidemiology [34]	Composite areas	Protocols submitted to Research Ethics Committee of the University Hospital Bern (Inselspital), Switzerland	CENTRAL database (Cochrane Library)	Protocols: from 1988 to 1998; Full reports: from 1990 to 2008	227/333	RCTs	Outcome reporting	Switzerland	Median sample size 330	Academic funding
Hahn, 2002, Journal of Evaluation in Clinical Practice [20]	Composite areas	Protocols from a local research ethics committee	Contacted the lead researcher	Not reported	37/15	Clinical trials	Selective reporting including study samples, outcomes, statistical plans, and result interpretations	Not reported	Not reported	Not reported
Dekkers, 2015, Journal of Clinical Epidemiology [11]	Composite areas	Protocols submitted to three research ethics committees in Switzerland and The Netherlands^a^	MEDLINE via PubMed, the Cochrane Controlled Trials Register and Google Scholar	Protocols: 2001 to 2005. Full reports: up to September 2012	54/54	Non-inferiority RCTs	Noninferiority margins	Switzerland and The Netherlands	Not reported	Academic funding
		Clinical Trials Registry Platform of the World Health Organization	MEDLINE via PubMed, the Cochrane Controlled Trials Register and Google Scholar	Registrations: up to March 2013. Full reports: up to September 2012	29/54					
Saquib, 2013, British Medical Journal [41]	Composite areas	Registration entries, protocols obtained by personal communication, and by searching design papers	Publications in PubMed in 25 biomedical journals with the highest impact factor	Full reports: publications published in 2009	162/199	RCTs	Statistical adjustments for primary outcomes	Not reported	Not reported	No funding received
Riveros, 2013, Plos Medicine [37]	Composite areas	Clinicaltrials.gov	Medline via PubMed	Registrations: up to March 2012. Full reports: up to March 2012	600/202	RCTs	Selective and incomplete outcome reporting	Not reported	Not reported	Academic funding
Boonacker, 2011, American Journal of Epidemiology [18]	Composite areas	Grant applications awarded by the Health Care Efficiency Research Program of the Netherlands Organization for Health Research and Development (ZonMw)	Pubmed	Grant applications: from 2001 to March 2010; Full reports: up to June 2010	79/79	RCTs and observational studies	Subgroup analyses	The Netherlands	Not reported	No funding received
Rising, 2008, PLOS Medicine [36]	Composite areas	FDA (Food and Drug Administration) reviews of approved NDAs (New Drug Applications) for new molecular entities (NMEs)	PubMed and The Cochrane Library	FDA reviews: from January 2001 to December 2002. Full reports: from July 2006 through June 2007	128/164	Clinical trails	Outcome reporting, statistical plan and conclusion	Not reported	Median sample size: 392	Academic funding
Hannink, 2013, Annals of Surgery [21]	Surgery	ClinicalTrials.gov, the International Standard Randomised Controlled Trial Number Registry (ISRCTN), Australian New Zealand Clinical Trials Registry (ANZCTR), or otherregistry	PubMed in 10 general medical journals and 10 surgical journals with the highest impact factors	Registrations: between 2007 and 2012. Full reports: between 2007 and 2012	152/152	RCTs	Outcome reporting	Not reported	Not reported	Not reported
Killeen, 2014, Annals of Surgery [24]	Surgery	Clinical trials.gov, ISRCTN, WHO trial registry portal, and the national/regional trial registry of the country of origin of the trial, and by e-mailing the corresponding author	The 10 surgical journals with the highest impact factor for 2009	Registrations: from 2009 to 2010. Full reports: from 2009 to 2010	108/108	RCTs	Outcome reporting	Not reported	Not reported	Not reported
Rongen, 2016, The Journal of Bone and Joint Surgery [38]	Orthopaedics	From the published articles, and by manually searching the corresponding trial registry and/or corresponding number	PubMed in the ten orthopaedic journals with the highest impact factors	Registrations: from January 2010 through December 2014. Full reports: from January 2010 through December 2014	26/26	RCTs	Primary outcome reporting	Not reported	Not reported	No funding received
Rosenthal, 2013, Annals of Surgery [40]	Surgery	Clinicaltrials.gov, the ISRCTN registry, and the WHO International Clinical Trials Registry Platform	*The Annals of Surgery, Archives of Surgery, and British Journal of Surgery*	Registrations and full reports: from January 2010, and December 2010	51/51	RCTs	Study sample, intervention, outcome reporting, statistical plan, study design, time duration, and funding/sponsor/ethics approval	Not reported	Not reported	No funding received
Vedula, 2009, The New England Journal of Medicine [45]	Pharmacotherapeutic studies (for gabapentin)	Internal company documents from Parke-Davis and Pfizer	Publications for trials sponsored by Parke-Davis and Pfizer	Protocols: partly from internal company documents, up to 2008. Full reports: up to 2008	20/12	Clinical trials	Outcome reporting	Not reported	Not reported	Litigation-related consultant fees
Vedula, 2013, PLOS Medicine [10]	Pharmaco therapeutic studies (for gabapentin)	Internal company documents from Parke-Davis and Pfizer	Publications for trials sponsored by Parke-Davis and Pfizer	Protocols: partly from internal company documents, up to 2008. Full reports: up to 2008	21/11	Clinical trials	Analysis reporting	Not reported	Not reported	Litigation-related consultant fees
Norris, 2014, Res. Syn. Meth [33]	Pharmacotherapeutic studies	International Clinical Trials Registry Platform	Publications of trials included in three comparative effectiveness reviews	Registrations: from January 2005 to January 2012. Full reports: from January 2005 to January 2012	50/50	RCTs	Selective outcome reporting and selective analysis reporting	Not reported	Not reported	Academic funding
Melander, 2003, British Medical Journal [29]	Pharmaco therapeutic study (for selective serotonin reuptake inhibitors)	For short term (4–8 weeks) RCTs with approved doses, applications that were submitted to the Swedish drug regulatory authority for marketing authorization and that included all relevant information	Medline (PubMed), Embase, and PsycINFO (Psychological Abstracts)	Applications: between 1983 and 1999. Full reports: between 1983 and 1999	42/38	Clinical trials	Analysis reporting	Sweden	Not reported	No funding received
Soares, 2004, British Medical Journal [48]	Oncology	Protocols for terminated RCTs conducted by Radiation Therapy Oncology Group	Publications of terminated RCTs conducted by Radiation Therapy Oncology Group	Protocols and full reports: from 1968 onward	56/58	RCTs	Study design, statistical plan, result presentation	USA and Canada	Not reported	Academic funding
Vera-Badillo, 2013, Annals of Oncology [46]	Oncology (for breast cancer)	ClinicalTrials.gov	PubMed	Registrations and full reports: from January 1995 to August 2011	30/164	RCTs	Outcome reporting	Not reported	Not reported	Not reported
Korevaar, 2014, Clinical Chemistry [25]	Diagnostic testing	ClinicalTrials.gov	PubMed, EMBASE, and Web of Science and by contacting investigators	Protocols: between January 2006 and December 2010. Full reports: up to 2013	418/153	Clinical trials	Inclusion criteria, results presentations and outcome reporting	Most in USA and Europe	Median sample size: 172	Not reported
Li, 2013, Scandinavian Journal of Gastroenterology [26]	Gastroenterology and hepatology	From the published article, and by searching the articles in the registries	PubMed search in top five general and internal journals and top five gastroenterology and hepatology journals	Registrations: from January 2009 to December 2012. Full reports: from January 2009 to December 2012	252/305	RCTs	Primary outcome reporting	46% in Europe	Median sample size: 388 in general and internal journals; 107 in gastroenterology and hepatology journals	No funding received
Mathieu, 2009, Journal of the American Medical Association [27]	Cardiology, rheumatology, and gastroenterology	From the published article, or corresponding author, or by searchingClinicalTrials.gov, ISRCTN registry, the registry of the country of the first or corresponding author, and World Health Organization (WHO) registry search portal	MEDLINE via PubMed and the 10 general medical journals and specialty journals with the highest impact factors	Registrations: up to March 2009. Full reports: up to March 2009	147/323	RCTs	Primary outcome reporting	Not reported	Not reported	Academic funding
Maund, 2014, British Medical Journal [28]	Major depressive disorder	Applications (including protocols and clinical study reports) submitted to European Medicines Agency (EMA) for study approvaland evaluation; registrations from Clinicaltrials.gov	PubMed and Cochrane Central Register of Controlled Trials, and by contacting the manufacturer	Applications and registrations: up to March 2013. Full reports: up to March 2013	9/7	RCTs	Results presentations and interpretations	Not reported	2878 in total	Academic funding
Milette, 2011, Journal of Psychosomatic Research [31]	Psychosomatic and behavioral health	From the published article, or corresponding author, or by searchingClinicalTrials.gov, ISRCTN registry, the registry of the country of the first or corresponding author. and World Health Organization registry search portal	Publications in PubMed in *Annals of Behavioral Medicine*, *Health Psychology*, *Journal of Psychosomatic Research*, and *Psychosomatic Medicine*	Registrations: from January 2008 to September 2009. Full reports: from January 2008 to September 2009	13/63	RCTs	Outcome reporting	Not reported	Nor reported	Nor reported
Nankervis, 2012, Journal of Investigative Dermatology [32]	Dermatology	WHO International Clinical Trial Registry Platform	Global Resource of Eczema Trials (GREAT) database	Registrations: from January 2007 to July 2011. Full reports: from January 2007 to July 2011	37/109	RCTs	Outcome reporting	Not reported	Median sample size: 70 for registered trials, 60 for unregistered trials	Academic funding
Riehm, 2015, Journal of Psychosomatic Research [35]	Psychosomatic and behavioral health	From the published article, or corresponding author, or by searchingClinicalTrials.gov, ISRCTN registry, the registry of the country of the first or corresponding author. and World Health Organization registry search portal	Publications in PubMed in *Annals of Behavioral Medicine*, *Health Psychology*, *Journal of Psychosomatic Research*, and *Psychosomatic Medicine*	Registrations: from January 2013 to October 2014. Full reports: from January 2013 to October 2014	40/76	RCTs	Outcome reporting	Not reported	Not reported	No funding
Rosati, 2016, Trials [39]	Pediatrics	Clinical trial registries including the United States National Institute of Health (clinicaltrials.gov), the ISRCTN registry, the Nederlands Trials Register (NTR), the Australian and New Zealand Clinical Trial Registry (ACTRN), and the Clinical Trial Registry-India (CTRI)	20 consecutive RCTs published from July to November 2013 in the journal *Pediatrics*	Registrations: from July 2013 to November 2013. Full reports: from July 2013 to November 2013	20/20	RCTs	Outcome reporting, sample size, statistical plan, result interpretation	Most conducted in USA	Not reported	No funding received
Su, 2015, Trials [43]	Acupuncture	15 major international trial registries	PubMed and three Chinese databases	Registrations and full reports: up to January 2014	88/96	RCT	Outcome reporting, inclusion criteria, and comparator	Most conducted in western countries (74%)	Sample size ranged from 10 to 960	Not reported
Turner, 2012, PLoS Medicine [44]	Antipsychotic Trials	FDA reviews on eight second-generation antipsychotic drugs for the treatment of schizophrenia	PubMed and Cochrane Central Register of Controlled Trials	FDA reviews: from December 1993 to May 2005. Full reports: up to May 2010	24/20	Clinical trials	Outcome reporting, result presentation	Not reported	Not reported	Academic funding
*Systematic review (n = 4)*										
Mhaskar, 2012, Journal of Clinical Epidemiology [30]	Oncology	Protocols of trials conducted by eight National Cancer Institute Cooperative Groups	Publications of trials conducted by eight National Cancer Institute Cooperative Groups	Protocols and full reports: Up to 2006	429/429	RCTs (Phase III)	Study sample, comparator, outcome, study design, statistical plan and randomization	Not reported	Not reported	Academic funding
You, 2012, Journal of clinical oncology [47]	Oncology	ClinicalTrials.gov and ISRCTN registry	MEDLINE via PubMed in 10 journals	Registrations: from August 2009 to April 2010. Full reports: from January 2005 to December 2009	215/366	RCTs	Outcome reporting	Not reported	Not reported	Academic funding
Hernandez, 2005, Neurosurgery [23]	Surgery (for traumatic brain injury)	Not reported	MEDLINE, EMBASE, the Cochrane Controlled Trials Register and the Cochrane Database of Systematic Reviews for RCTs	Full reports: up to April 2004	6/6	RCTs	Subgroup analyses	Most in USA and Europe	Median sample size: 463	Not reported
Smith, 2013, Pain [42]	Analgesic studies	ClinicalTrials.gov	Registered Analgesic Clinical Trials (RReACT) database	Full reports: Trials registered at ClinicalTrials.gov as of December 2011	87/87	Clinical Trials	Outcome reporting	Not reported	Median sample size: 167	Not reported

*RCT* randomized controlled trial

^a^Ethics committees including KantonaleEthikkommission Bern; Commission d’ethique de la recherche sur l’etrehumain; and EthischeCommissieLeidsUniversitairMedisch Centrum

^b^Indicated comparisons (protocols vs. full reports, or registrations vs. full reported) were highlighted in this column

We assessed study quality for the four systematic reviews using AMSTAR [23, 30, 42, 47]. None of them had assessed the quality of their included primary studies, thus receiving no points for items 7 (“Was the scientific quality of the included studies assessed and documented?”) and 8 (“Was the scientific quality of the included studies used appropriately in formulating conclusions?”). One review scored 5 (out of 9) on AMSTAR, because it did not provided information on duplicate data collection (AMSTAR item 2) or show the list of included and excluded primary studies (item 5) [23]. No indication of a grey literature search (item 4) was found in one systematic review [42], resulting in its score of 6 (out of 9) on AMSTAR.

**Fig. 2:**
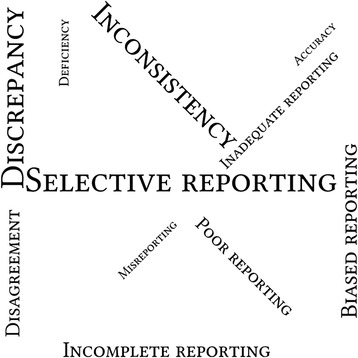
Word clouds of the terminologies used in the included studies, with the relative size of the terms in the word clouds corresponding to the frequency of their use

Among all the 37 included studies, the terminologies most frequently used to describe the reporting problem included *selective reporting* (*n* = 35, 95%), *discrepancy* (*n* = 31, 84%), *inconsistency* (*n* = 27, 73%), *biased reporting* (*n* = 15, 41%), and *incomplete reporting* (*n* = 13, 35%). Fig. 2 shows the word clouds of all the terminologies used in the included systematic reviews and surveys.

Table 2 presents the key findings and authors’ conclusions of inconsistency by their main measure of comparison between protocols or registrations and full reports. Table 3 presented the detailed information of what had been reported in the 37 included studies regarding the inconsistency between protocols or registrations and full reports. There were 17 studies with a focus of **outcome reporting** problems, including changing, omitting (or unreported), introducing, incompletely-reporting, and selectively-reporting outcomes. The median inconsistency of outcome reporting was 54% (IQR: 29% - 72%), ranging from 14% (22/155) to 100% (1/1 and 69/69). Six studies found that most inconsistencies (median 71%, IQR: 57% - 83%) favoured a statistically significant result in full reports [21, 24, 27, 38, 43, 45]. Regarding **subgroup reporting**, inconsistency levels between protocols or registrations and full reports varied from 38% (196/515) to 100% (6/6), with post hoc analyses introduced (ranging from 26% (132/515) to 76% (143/189)) and pre-specified analyses omitted in full reports (from 12% (64/515) to 69% (103/149)). Inconsistencies of **statistical analyses** were observed, including defining non-inferiority margins, analysis principle selection (intention-to-treat, per-protocol, as-treated), and model adjustment, with an inconsistency level varying from 9% (5/54) to 67% (2/3). The remaining 13 studies reported frequent inconsistencies in **multiple measure comparisons**, where the multiple measure comparisons were defined as at least two main measures used for comparison between protocols or registrations and full reports (Tables 2 and 3). For instance, inconsistencies were observed in sample sizes (ranged from 27% (14/51) to 60% (34/56)), inclusion or exclusion criteria (from 12% (19/153) to 45% (9/20)), and conclusions (9%, 9/99).

**Table 2:** Key findings and authors’ conclusions of inconsistency by main measure of comparison between protocols or registrations and full reports in the included studies

First author, year	Key findings of inconsistent reporting	Authors’ conclusions
*Outcome reporting (n = 17)*		
Chan, 2004 (from CMAJ) [19]	88% (42/48) and 62% (16/26) trials had at least one unreported efficacy and harm outcome respectively. 96% (46/48) and 81% (21/26) trials had incompletely reported efficacy and harm outcome respectively.	“Selective reporting of outcomes frequently occurs in publications of high-quality government-funded trials.”
Chan, 2004 (from JAMA) [7]	50% (50/99) and 65% (47/72) trials had at least one incompletely reported efficacy and harm outcome respectively. 62% (51/82) trials had at least one primary outcome changed, omitted, or introduced.	“The reporting of trial outcomes in journals is frequently inadequate to provide sufficient data for interpretation and meta-analysis, is biased to favor statistical significance, and is inconsistent with primary outcomes specified in trial protocols. These deficiencies in outcome reporting pose a threat to the reliability of the randomized trial literature.”
Hannink, 2013 [21]	49% (75/152) showed some discrepancies in outcomes, most related to introducing or omitting a primary outcome. 28% (21/75) of theses discrepancies favored statistically significant results.	“Comparison of the primary outcomes of surgical RCTs registered with their subsequent publication indicated that selective outcome reporting is (highly) prevalent and appears to be more common in surgical trials than in general medical trials.”
Hartung, 2014 [22]	80% (88/110) trials reported the number of secondary outcome measures inconsistently. 15% (16/110) reported definition of primary outcome measures inconsistently; 20% (22/110) reported results of primary outcome measures inconsistently. 35% (38/110) reporting the number of participants with a serious adverse event (SAE) inconsistently; of these, 87% (33/38) results in ClinicalTrials.gov reported more SAEs.	“Reporting discrepancies between the ClinicalTrials.gov results database and matching publications are common. Which source contains the more accurate account of results is unclear, although ClinicalTrials.gov may provide a more comprehensive description of adverse events than the publication.”
Killeen, 2014 [24]	29% (32/108) registered trials had a discrepancy of primary outcomes between registrations and full reports. 92% of the discrepancies in primary outcomes (in 22 out of 24 full reports) favored a statistically significant finding.	“Less than half of all RCTs published in general surgical journals were adequately registered, and approximately 30% had discrepancies in the registered and published primary outcome with 90% of those assessable favoring a statistically positive result.”
Li, 2013 [26]	14% (22/155) RCTs had discrepancies in primary outcomes between registrations and full reports.	“Based on the results of the present study, selective outcome reporting of gastroenterology RCTs published in leading medical journals has been much improved over the past years. However, there might be a sampling bias to say that consistency of registered and published POs of gastroenterology RCTs has been better than before.”
Mathieu, 2009 [27]	31% (46/147) full reports had discrepancies in outcomes compared with registrations. 83% of the discrepancies (in 19 out of 23 full reports) favored a statistically significant result.	“Comparison of the primary outcomes of RCTs registered with their subsequent publication indicated that selective outcome reporting is prevalent.”
Milette, 2011 [31]	21% (13/63) full reports were registered; only one trial (8%, out of 13) could provide sufficient information to compare full reports with registration for outcomes, and discrepancies (100%) in outcome was found in the study.	“Greater attention to outcome reporting and trial registration by researchers, peer reviewers, and journal editors will increase the likelihood that effective behavioral health interventions are readily identified and made available to patients.”
Nankervis, 2012 [32]	17% (18/109) full reports were properly registered. 72% (13/18) full reports had inconsistencies compared with registrations.	“Adequate trial registration for eczema RCTs is poor. Registration of all trials in a publicly accessible database is a critical step toward ensuring the transparent reporting of clinical trial results that affect health care.”
Redmond, 2013 [34]	29% outcomes (870/2966) reported inconsistently. 7% (19/274) primary outcomes in protocols not reported in full reports; 10% (30/288) primary outcomes reported in full reports but not found in protocols. 19% (284/1495) secondary outcomes in protocols not reported in full reports; 14% (334/2375) secondary outcomes reported in full reports but not found in protocols.	“Discrepant reporting was associated with statistical significance of results, type of outcome, and specialty area. Trial protocols should be made freely available, and the publications should describe and justify any changes made to protocol-defined outcomes.”
Riehm, 2015 [35]	Only 3 out of 40 studies were registered; discrepant outcomes were found in 1 of these 3 studies (33%).	“The quality of published outcome declarations and trial registrations remains largely inadequate. Greater attention to trial registration and outcome definition in published reports is needed.”
Rongen, 2016 [38]	25% (90/362) full reports were registered. 54% (14/26) full reports had one or multiple major discrepancies with registrations, 57% (8/14) of which favored statistically significant findings.	“Although trial registration is now the rule, it is currently far from optimal for orthopaedic surgical RCTs and selective outcome reporting is prevalent. Full involvement of authors, editors, and reviewers is necessary to ensure publication of quality, unbiased results.”
Smith, 2013 [42]	100% (69/69) full reports had discrepancies in primary outcome specifications (POS). 30% (21/69) full reports had unambiguous POS discrepancies, with significantly higher percentages of non-industry-sponsored than industry-sponsored full reports having unambiguous POS discrepancies.	“At best, POS discrepancies may be attributable to insufficient registry requirements, carelessness (eg, failing to report PO assessment timing), or difficulty uploading registry information. At worst, discrepancies could indicate investigator impropriety (eg, registering imprecise PO [“pain”], then publishing whichever pain assessment produced statistically significant results). Improvements in PO registration, as well as journal policies requiring consistency between registered and published PO descriptions, are needed.”
Su, 2015 [43]	19% (17/88) full reports were registered. 45% (32/71) full reports had inconsistency of primary outcomes; 71% (15/21) had discrepancies in primary outcomes that favored significant findings.	“We find that prospective registration for randomized clinical trials on acupuncture is insufficient, selective outcome reporting is prevalent, and the change of primary outcomes is intended to favor statistical significance. These discrepancies in outcome reporting may lead to biased and misleading results of randomized clinical trials on acupuncture. To ensure publication of reliable and unbiased results, further promotion and implementation of trial registration are still needed.”
Vedula, 2009 [45]	67% (8/12) full reports reported primary outcomes differently from internal company documents. Primary outcomes in internal company documents with nonsignificant results were either unreported in full reports, or were reported with a changed outcome measure.	“Selective outcome reporting was identified for trials of off-label use of gabapentin. This practice threatens the validity of evidence for the effectiveness of off-label interventions.”
Vera-Badillo 2013 [46]	18% (30/164) full reports were registered; of these, 23% (7/30) had a changed primary outcome measure compared with registrations.	“Bias in the reporting of efficacy and toxicity remains prevalent. Clinicians, reviewers, journal editors and regulators should apply a critical eye to trial reports and be wary of the possibility of biased reporting. Guidelines are necessary to improve the reporting of both efficacy and toxicity.”
You, 2012 [47]	14% (19/134) full reports had inconsistency in primary end points (PEPs) compared with registrations.	“The rates of trial registration and of trials with clearly defined PEPs have improved over time; however, 14% of these trials reported a different PEP in the final publication. Intrapublication inconsistencies in PEP reporting are frequent.”
*Subgroup reporting (n = 3)*		
Boonacker, 2011 [18]	75% (59/79) full reports had differences in subgroup analyses from grant applications. 69% prespecified subgroup analyses (103/149) were not reported in full reports. 76% subgroup analyses (143/189) were based on post hoc results.	“There is a large discrepancy between the grant applications and the final publications regarding subgroup analyses. Both nonreporting prespecified subgroup analyses and reporting post-hoc subgroup analyses are common. More guidance is clearly needed.”
Hernandez, 2005	100% (6/6) full reports had discrepancies in subgroup analyses from protocols.	“The reported covariate adjustment and subgroup analyses from TBI trials had several methodological shortcomings. Appropriate performance and reporting of covariate adjustment and subgroup analysis should be considerably improved in future TBI trials because interpretation of treatment benefits may be misleading otherwise.”
Kasenda, 2014 [9]	26% (132/515) trials reported the subgroup analyses that were not mentioned in their protocols. 12% (64/515) trials did not reported subgroup analyses that were planned in their protocols.	“Large discrepancies exist between the planning and reporting of subgroup analyses in RCTs. Published statements about subgroup prespecification were not supported by study protocols in about a third of cases. Our results highlight the importance of enhancing the completeness and accuracy of protocols of RCTs and their accessibility to journal editors, reviewers, and readers.”
*Statistical analysis reporting (n = 4)*		
Dekkers, 2015 [11]^a^	Noninferiority margin was inconsistently reported (9%, 5/54 trials), or not reported in the full reports (9%, 5/54), or not defined in the protocol (2%, 1/54). Reporting of both noninferiority margin and confidence interval (or *p*-value) was incomplete or inconsistent (28%, 15/54). 54% (29/54) trials were registered, but only one registry record (3%, 1/29) provided information on noninferiority margin.	“The reporting of noninferiority margins was incomplete and inconsistent with study protocols in a substantial proportion of published trials, and margins were rarely reported in trial registries.”
Melander, 2003 [29]	98% (41/42) documents submitted to regulatory authority provided two or more analyses (intention-to-treat, and per-protocol analysis). 7% (2/28) full reports based on a single trial (stand alone publications) provided an intention-to-treat as well as per-protocol analysis; the remaining stand alone publications (93%, 26/28) only provided one analysis that tended to be per-protocol analysis. 20 full reports (15 stand alone publications, and 5 pooled publications that were based on two or more trials) showed difference in participant response rates compared with documents submitted to regulatory authority.	“The degree of multiple publication, selective publication, and selective reporting differed between products. Thus, any attempt to recommend a specific selective serotonin reuptake inhibitor from the publicly available data only is likely to be based on biased evidence.”
Saquib, 2013 [41]	6% (9/162) trials had statistical analyses such as model adjustments described in registrations, 78% (21/27) in design papers, and 74% (40/54) in protocols obtained from authors. 47% (28/60) full reports had discrepancies in analyses plans compared with registrations, protocols or design papers.	“There is large diversity on whether and how analyses of primary outcomes are adjusted in randomized controlled trials and these choices can sometimes change the nominal significance of the results. Registered protocols should explicitly specify adjustments plans for main outcomes and analysis should follow these plans.”
Vedula, 2013 [10]	Intention-to-treat analyses were defined differently between internal company documents and full reports, resulting in different number of participants in analyses and different results.	“Descriptions of analyses conducted did not agree between internal company documents and what was publicly reported. Internal company documents provide extensive documentation of methods planned and used, and trial findings, and should be publicly accessible. Reporting standards for RCTs should recommend transparent descriptions and definitions of analyses performed and which study participants are excluded.”
*Multiple measure comparison* ^*b*^ *(n = 13)*		
Al-Marzouki, 2008 [17]	30% (11/37) trials had major discrepancy between protocols and full reports for primary outcomes: 5 had an unreported primary outcome; 8 introduced a new primary outcome; 2 changed a primary outcome to secondary 49% (18/37) trials mentioned subgroup analyses in the protocols; but 76% (28/37) reported subgroup analyses. Only one protocol (3%) provided reasons for the subgroup choice.	“Although the solution to the problem of selective reporting requires further discussion, the current system is clearly inadequate.”
Chan, 2008 [8]	Unacknowledged differences between protocols and full reports were observed in sample size calculation (53%, 18/34 trials), methods of handling protocol deviation (44%, 19/43), addressing missing data (80%, 39/49), primary outcome analyses (60%, 25/42), subgroup analyses (100%, 25/25), adjusted models (82%, 23/28), and interim analyses (62%, 8/13).	“When reported in publications, sample size calculations and statistical methods were often explicitly discrepant with the protocol or not pre-specified. Such amendments were rarely acknowledged in the trial publication. The reliability of trial reports cannot be assessed without having access to the full protocols.”
Hahn, 2002 [20]	60% (9/15) trials did not state primary outcomes. 47% (7/15) did not mentioned analysis plans. In the 8 trials mentioning analysis plans, 88% (7/8) did not follow the prespecified plans.	“This pilot study has shown that within-study selective reporting may be examined qualitatively by comparing the study report with the study protocol. The results suggest that it might well be substantial; however, the bias can only be broadly identified as protocols are not sufficiently precise.”
Korevaar, 2014 [25]	32% (49/153) full reports had discrepancies compared with registrations: 12% (19/153) had discrepancies in inclusion criteria; 6% (9/153) in result presentations, and 21% (32/153) in outcomes	“Failure to publish and selective reporting are prevalent in test accuracy studies. Their registration should be further promoted among researchers and journal editors.”
Maund, 2014 [28]	Minor inconsistencies in population in the primary efficacy analysis found in one trial (out of 7) between protocol and full report and within the full report. Incomplete reporting of adverse events found in full reports.	“Clinical study reports contained extensive data on major harms that were not available in journal articles and in trial registry reports. There were minor inconsistencies in primary efficacy analysis population between protocols and clinical study reports and within clinical study reports. There were also inconsistencies between different summaries and tabulations of harms data within clinical study reports. Clinical study reports should be used as the data source for systematic reviews of drugs, but they should first be checked against protocols and within themselves for accuracy and consistency.”
Mhaskar, 2012 [30]	Overall methodological quality reporting in full reports was poor and did not reflect actual high quality in protocols.	“The largest study to date shows that poor quality of reporting does not reflect the actual high methodological quality. Assessment of the impact of quality on the effect size based on reported quality can produce misleading results.”
Norris, 2014 [33]	90% (45/50) full reports had selective outcome reporting (SOR) or selective analysis reporting (SAR) compared with their registrations.	“The SOR and SAR were frequent in this pilot study, and the most common type of SOR was the publication of outcomes that were not pre-specified. Trial registries were of little use in identifying SOR and of no use in identifying SAR.”
Rising, 2008 [36]	41 primary outcomes from FDA reviews of applications were omitted from full reports; 15 outcomes were added in full reports that favored the drug tested. 43 outcomes in FDA reviews that did not favor the drug tested; of these, 20 (47%) were omitted from full reports; 5 of the remaining 23 outcomes changed in full reports, with 4 (80%, out of 5) changing to favor the drug tested in full reports. 99 conclusions provided in both FDA reviews and full reports; of these, 9% conclusions (9/99) changed from FDA reviews to full reports so that they favored the drug tested in full reports.	“Discrepancies between the trial information reviewed by the FDA and information found in published trials tended to lead to more favorable presentations of the NDA drugs in the publications. Thus, the information that is readily available in the scientific literature to health care professionals is incomplete and potentially biased.”
Riveros, 2013 [37]	More complete reporting was found in registry than in full reports for selection flow of participants (64% vs 48%), efficacy findings (79% vs 69%), adverse events (73% vs 45%), and serious adverse events (99% vs 63%).	“Our results highlight the need to search ClinicalTrials.gov for both unpublished and published trials. Trial results, especially serious adverse events, are more completely reported at ClinicalTrials.gov than in the published article.”
Rosati, 2016 [39]	95% (19/20) full reports had medium or high combined discrepancy scores comparing registrations. 100% (20/20) full reports selectively reported or unreported main outcomes; 45% (9/20) had discrepancies in disclosing funding, 40% (8/20) in sample size, 45% (9/20) in inclusion or exclusion criteria, 55% (11/20) changed primary outcome to secondary (or vice versa), and 65% (13/20) discontinued early with no justifications in full reports.	“Major discrepancies between what clinical trial registrations record and paediatric RCTs publish raise concern about what clinical trials conclude. Our findings should make clinicians, who rely on RCT results for medical decision-making, aware of dissemination or reporting bias. Trialists need to bring CTR data and reported protocols into line with published data.”
Rosenthal, 2013 [40]	22% (11/51) full reports downgraded primary outcomes (defined by registrations) as secondary; 8% (4/51) completely omitted primary outcomes; 8% (4/51) introduced a new primary outcome, and 10% (5/51) defined primary outcome differently. Few discrepancies in randomization, blinding, intervention and ethical committee approval, and some in sample size and inclusion or exclusion criteria. 45% (23/51) full reports had funding information that was not in registrations.	“When interpreting the results of surgical RCTs, the possibility of selective reporting, and thus outcome reporting bias, has to be kept in mind. For future trials, prospective registration should be strictly respected with the ultimate goal to increase transparency and contribute to high-level evidence reports for optimal patient care in surgery.”
Soares, 2004 [48]	The methodological quality in 56 full reports was worse than in protocols. Only 42% reported allocation concealment (while all protocols achieved allocation concealment); 69% reported intention-to-treat analysis (while 83% protocols did such analysis); 16% reported sample size calculation (while 76% protocols did so); 10% reported endpoints and errors (while 76% and 74% protocols defined endpoints and errors respectively).	“The reporting of methodological aspects of RCTs does not necessarily reflect the conduct of the trial. Reviewing research protocols and contacting trialists for more information may improve quality assessment.”
Turner, 2012 [44]	17% FDA-registered trials not published (4 trials out of 24 applications). 25% (5/20) full reports did not have positive findings Effect size for unpublished trials (0.23) was significantly less than that for published full reports (effect size: 0.47).	“The magnitude of publication bias found for antipsychotics was less than that found previously for antidepressants, possibly because antipsychotics demonstrate superiority to placebo more consistently. Without increased access to regulatory agency data, publication bias will continue to blur distinctions between effective and ineffective drugs.”

^a^This study focused on noninferiority margin reporting

^b^Multiple measure comparison defined as at least two main measures used for comparisons, including comparisons of participant, outcome, subgroup, analysis, result, effect size, inclusion criteria, sample size, control, randomization, blinding, intervention, funding, ethics, and/or conclusion reporting

**Table 3:** Detailed information of what had been reported in the included studies regarding the inconsistency between protocols or registrations and full reports^a^

First author, year	Levels of inconsistent reporting between protocols or registrations and full reports										
	Participant eligibility criteria	Sample size	Randomization	Interventions and their delivery	Blinding	Outcome measure	Study duration	Statistical analysis choice (e.g., model selection, model adjustment, missing data handling, intention-to-treat, et al)	Subgroup analysis	Funding source	Others^b^
Al-Marzouki, 2008 [17]	–	–	–	–	–	30%	–	–	36%	–	–
Chan, 2004 (from CMAJ) [19]	–	–	–	–	–	40%	–	–	–	–	–
Chan, 2004 (from JAMA) [7]	–	–	–	–	–	62%	–	–	–	–	–
Boonacker, 2011 [18]	–	–	–	–	–	–	–	–	75%	–	–
Chan, 2008 [3]	–	53%	–	–	–	60%	–	Handling missing data: 80% Interim analyses: 62% Model adjustments: 82%	100%	–	
Dekkers, 2015 [11]	–	–	–	–	–	–	–	Noninferiority margin definitions reported: 9% Noninferiority margins and confidence intervals reported: 28%	–	–	–
Hahn, 2002 [20]	–	–	–	–	–	33%	–	Analysis plans reported: 88%	–	–	–
Hannink, 2013 [21]	–	–	–	–	–	49%	–	–	–	–	–
Hartung, 2014 [22]	–	–	–	–	–	15%	–	–	–	–	
Hernandez, 2005 [23]	–	–	–	–	–	–	–	–	100%	–	–
Kasenda, 2014 [9]	–	–	–	–	–	–	–	–	38%	–	–
Killlen, 2014	–	–	–	–	–	29%	–	–	–	–	–
Korevaar, 2014 [25]	12%	–	–	–	–	21%	–		–	–	Result presentations: 6%
Li, 2013 [26]	–	–	–	–	–	14%	–	–	–	–	–
Mathieu, 2009 [27]	–	–	–	–	–	31%	–	–	–	–	–
Maund, 2014 [28]	–	–	–	–	–	–	–	Primary efficacy analyses reported: 14%	–	–	Adverse events reported: 100%
Melander, 2003 [29]	–	–	–	–	–	–	–	Intention-to-treat or per-protocol analyses reported: 93%	–	–	–
Mhaskar, 2012 [30]	–	59%	77%	72%	53%	–	–	Intention-to-treat analyses reported: 70%	–	–	
Milette, 2011 [31]	–	–	–	–	–	100%	–	–	–	–	–
Nankervis, 2012 [32]	–	–	–	–	–	72%	–	–		–	–
Norris, 2014 [33]	–	–	–	–	–	83%	–	–	20%	–	Adverse events reported; 43%
Redmond, 2013 [34]	–	–	–	–	–	29% (primary outcomes: 17%; secondary outcomes: 33%)	–	–	–	–	–
Riehm, 2015 [35]	–	–	–	–	–	33%	–	–	–	–	–
Rising, 2008 [36]	–	–	–	–	–	31%	–	–	–	–	Conclusions reported: 9%
Riveros, 2013 [37]	–	–	–	–	–	87%	–	–	–	–	Adverse events reported: 37%
Rongen, 2016 [38]	–	–	–	–	–	54%		–	–	–	–
Rosati, 2016 [39]	45%	40%	–	–	–	100%	Studies discontinued early without justifications:65%	Intention-to-treat analysis: 10%	–	45%	
Rosenthal, 2013 [40]	–	27%	2%	–	23%	Primary outcomes: 45% Secondary outcomes: 67%	Start of patient enrolment reported: 43% End of enrollment reported: 71%	–	–	45%	Ethical committee approval: 2%
Saquib, 2013 [41]	–	–	–	–	–	–	–	Analysis plans reported: 47%	–	–	–
Smith, 2013 [42]	–	–	–	–	–	79%	–	–	–	–	–
Soares, 2004 [48]	–	60%	–	59%	–	–	–	Intention-to-treat analysis: 14%	–	–	Dropouts reported: 9%
Su, 2015 [43]	–	–	–	–	–	45%	–	–	–	–	–
Turner, 2012 [44]	–	–	–	–	–	25%-	–	–	–	–	Effect sizes reported: 8%
Vedula, 2009 [45]	–	–	–	–	–	67%	–	–	–	–	–
Vedula, 2013 [10]	–	–	–	–	–	–	–	Intention-to-treat analysis definitions: 67% Safety analysis definitions: 50%	–	–	–
Vera-Badillo 2013 [46]	–	–	–	–	–	23%	–	–	–	–	–
You, 2012 [47]	–	–	–	–	–	14%	–	–	–	–	–

^a^Cells with a ‘-’ indicated the information was not reported in the included studies

^b^Other inconsistency measures including comparisons of effect size, sample size, control, ethics, key finding reporting, and/or conclusion reporting

As shown in Table 4, significant factors reported to be related to inconsistent reporting included outcomes with statistically significant results, study sponsorship, type of outcome (efficacy, harm outcome) and disease specialty. Two studies reported higher odds of complete reporting for full reports in primary outcomes with significant results (odds ratios (ORs) ranging from 2.5 to 4.7) [7, 19], while one study found that outcomes with significant results were associated with inconsistent reporting in full reports (OR = 1.38) [34].Other factors related to inconsistent reporting included investigator-sponsored trials, efficacy outcomes, and cardiology and infectious diseases (Table 4).

**Table 4:** Significant factors reported to be related with inconsistency between protocols or registrations and full reports

First author, year	Main measures of comparison	Significant factors related with inconsistent reporting	Association between factors and inconsistent reporting
Chan, 2004 [7]	Outcome reporting	Outcomes with statistically significant results	Higher odds of being fully reported in primary efficacy outcomes with significant results, compared with primary efficacy outcomes with nonsignificant results (odds ratio [OR] = 2.7, 95% confidence interval [CI]: 1.5–5.0)
Chan, 2004 [7]	Outcome reporting	Outcomes with statistically significant results	Higher odds of being fully reported in primary efficacy outcomes with significant results, compared with primary efficacy outcomes with nonsignificant results (OR = 2.4, 95% CI: 1.4–4.0); corresponding odds ratio for primary harm outcomes was 4.7 (95% CI: 1.8–12.0)
Kasenda, 2014 [9]	Subgroup analysis	Study sponsorship	Subgroup analyses were more often planned in industry-sponsored trials, compared with investigator-sponsored trials (*p* < 0.001)
Redmond, 2013 [34]	Outcome reporting	Outcomes with statistically significant results; Efficacy outcomes (vs harm outcomes); Cardiology (vs all specialties); Infectious diseases (vs all specialties)	Higher odds of inconsistent reporting found in outcomes with significant results (OR = 1.38, 95% CI: 1.07–1.78), in efficacy outcomes compared with harm outcomes (OR = 2.99, 95% CI: 2.08–4.30), in Cardiology specialty (OR = 2.34, 95% CI: 1.44–3.79), and in Infectious diseases (OR = 1.77, 95% CI: 1.01–3.13) compared with all specialties combined.

## Discussion

We have presented the mapping of evidence of inconsistent reporting between protocols or registrations (i.e., *what was planned to be done* and/or *what was actually done*) and full reports (i.e., *what was reported in the literature*) in primary biomedical research, based on findings from systematic reviews and surveys in the literature. High levels of inconsistency were found across various areas in biomedicine and in different study aspects, including outcome reporting, subgroup reporting, statistical analyses, and others. Some factors such as outcomes with significant results, sponsorship, type of outcome and disease speciality were reported to be significantly related with inconsistent reporting.

The ICMJE statement that requires all trials to be registered prospectively has been implemented since 2004 [5]. Likewise, the SPIRIT (Standard Protocol Items: Recommendations for Interventional Trials) statement aims to assist in transparent reporting and improve the quality of protocols [49]. However, inconsistent reporting between protocols or registrations and full reports remains a severe problem. In this review, all the included studies revealed that the inconsistent reporting between protocols or registrations and full reports was highly prevalent, common and suboptimal. Inconsistent reporting may impair the evidence’s reliability and validity in the literature, potentially resulting in evidence-biased syntheses [29, 50, 51] and inaccurate decision-making, especially given that most inconsistencies were found to favor statistically significant results (Table 2).One study searched full reports in gastroenterology and hepatology journals published from 2009 to 2012, and concluded that the inconsistent reporting problem had improved; however, there might have been sampling bias involved in reaching this conclusion as it indicated [26]. More evidence to assess the trend of inconsistent reporting, and more efforts to mitigate it, are needed for the primary biomedical community.

We found that the majority of the evidence for inconsistent reporting between protocols or registrations and full reports came from assessments of outcome reporting. It is not uncommon for authors to change, omit, incompletely-report, selectively-report, or introduce new outcomes in full reports. The main reason was that they attempted to show statistically significant findings using an approach of selective reporting of outcomes to cater to the journal’s choices of publications [52–54].Therefore outcomes with significant results were more likely to be fully and completely reported, compared with those outcomes with nonsignificant results, as observed in two included surveys [7, 19]. By contrast, another study found that outcomes with significant results were associated with inconsistent reporting [34]. The conflicting findings may be due to their different inclusion criteria (primary outcomes [7, 19] vs. all outcomes [34]), and different definitions of inconsistent reporting (defined as primary outcomes changed, introduced, or omitted [7, 19] vs. defined as addition, omission, non-specification, or reclassification of primary and secondary outcomes [34]). Thus more research is needed to further explore and clarify the relationship between outcomes with significant results and inconsistent reporting. Similarly, other factors (study sponsorship, type of outcome and disease speciality) should be considered with caution, because their associations with inconsistent reporting were observed in only one survey (Table 4).

We identified several methodological issues in the included studies. Some studies used multiple sources to locate protocol or registration documents and full reports. However, we could not study the heterogeneity in the sources of protocols or registrations and full reports used for comparisons, because the sources used were substantially various and some of them could not be publicly accessible. Furthermore, although registrations are publicly available, they usually contain incomplete study information [55]. Protocols can provide more transparent and comprehensive details, but they are often not publicly accessible. This is a major limitation that may make it harder to reproduce the findings and conclusions of comparing protocols or registrations and full reports. Likewise, the definitions of inconsistencies and measures of the level of inconsistencies were not fully and explicitly described in the included studies, potentially impacting the reproducibility and validation of the evaluations. This challenge is further exacerbated by lack of detailed and transparent reporting of the data collection methods in some studies. For example, some surveys only contacted authors for access to full reports, rather than systematically searching the database(s). Such heterogeneity and disagreements across data sources would potentially affect the statistical significances, effect sizes, interpretations, and conclusions of trial results and their subsequent meta-analyses [56]. Also, there were no explanations provided regarding the inconsistencies found between documents. One study conducted a telephone interview with trialists who were identified to experience inconsistent reporting [57]. It was found that most trialists were not aware of the implications for the evidence base of inconsistent reporting in full trial reports. Thus, providing the researchers with some support to help them recognize the importance of consistent reporting, such as including a list of trial modifications as a journal requirement for submission and offering some training sessions with different inconsistent reporting scenarios that could drive different conclusions, would be a worthwhile endeavour. Taken together, these issues raise the importance of establishing appropriate standards for and consensus on conducting scientific studies aimed at comparing the reporting of key trial aspects in different documents so as to enhance the reproducibility of such comparison studies.

There were several systematic reviews assessing cohort studies that compared protocols or registrations and full reports [52, 53, 58, 59]. However, they either focused on outcome reporting [52, 53] or statistical analysis reporting [58]; and therefore there was no study summarizing all the inconsistencies between protocols or registrations and full reports in the primary research literature mapping. One Cochrane review published in 2011 included 16 studies and assessed all aspects of inconsistencies throughout the full reports [59]. Our current review included more up-to-date studies and thus provided more information for the biomedical community. Moreover, while all the reviews restricted their inclusion of clinical trials only [52, 53, 58, 59], our review aimed to include all the biomedical areas and map the existing evidence in the overall primary biomedical community. Furthermore, our study identified several methodological issues in the included systematic reviews and surveys regarding the design, conduct and reproducibility, which could assist with the transparent and standardized processes of future comparison studies in this topic.

With a high prevalence of inconsistent reporting highlighted in this review, efforts are needed to reverse this condition by authors, journals, sponsors, regulators and research ethics committees. For instance, authors are expected to fully interpret the necessary modifications made from protocols or registrations, while journal staff and reviewers should refer to protocols or registrations for rigorous scrutiny in peer-review processes. Moreover, the investigators who share their protocols, full reports, and data in public should be rewarded, because this practice can mitigate the inconsistent reporting problem and increase the scientific value of research [54]. For instance, institutions and funders might consider using some performance metrics to provide credits or promotions for the investigators who are willing to share and disseminate their research in public [54]. The impact of ICMJE and SPIRIT statements on inconsistent reporting remains largely unexplored due to sparse evidence available. However, such standards for the protocol or registration reporting should be strictly adopted for all the biomedical areas, because they can provide a platform for easy evaluation of and comparison with full reports. For example, some studies found that prospective registrations for trials were inadequate and incomplete [24, 25, 32, 35, 43], leaving the comparison between registrations and full reports questionable and unidentifiable. Therefore the possibility of inconsistent reporting remained largely unknown for those trials with inadequate and incomplete registrations, which would exert an unclear impact on our findings in this review. On the other hand, two studies demonstrated that the methodological quality in full reports was poor and could not reflect the actual high quality in protocols [30, 48]. Therefore to improve their quality of reporting and reduce the inconsistent reporting, full reports should rigorously follow the reporting guidelines including the CONSORT (Consolidated Standards of Reporting Trials) for clinical trials, ARRIVE (Animal Research: Reporting In Vivo Experiments) for animal studies, and STROBE (Strengthening the Reporting of Observational Studies in Epidemiology) for observational studies, among others. For instance, one study comparing the quality of trial reporting in 2006 between the CONSORT endorsing and non-endorsing journals found significantly improved reporting quality for the trials published in the CONSORT endorsing journals, especially for the aspect of trial registrations (risk ratio = 5.33; 95% confidence interval: 2.82 to 10.08) [60]. Besides, guidance and/or checklists are needed for authors, editorial staff, reviewers, sponsors, regulators and research ethics committees to advance their easy and prompt assessment of inconsistency between protocols or registrations and full reports.

Some limitations exist in this review. We limited our search to English language, which would restrict the generalizability of our findings to the studies in other languages. We did not search the grey literature for unpublished systematic reviews or surveys, which may omit the data from studies that were in progress or yet to be published. We only included one study exploring non-trial research (Table 1); therefore, the inconsistent reporting in non-trial areas remains largely unknown. A possible explanation for this may be that compared to trials, non-trial or observational studies continue to receive less scrutiny in that there is no requirement for their registration, and also there is less emphasis on publication of their protocols. We could not evaluate the quality of surveys due to lack of quality assessment guidance available, which would impair the strength of evidence presented in our review, because most included studies were surveys.

## Conclusion

In this systematic review comparing protocols or registrations with full reports, we highlight that inconsistent reporting in different study aspects is frequent, prevalent and suboptimal in primary biomedical research, based on findings from systematic reviews and surveys in the literature. We also identify methodological issues such as the need for consensus on measuring inconsistency across sources for trial reports, and more studies evaluating transparency and reproducibility in reporting all aspects of study design and analysis. Efforts from authors, journals, sponsors, regulators and research ethics committees are urgently required to reverse the inconsistent reporting problem.

## Abbreviations

AMSTAR: a measurement tool to assess systematic reviews
ARRIVE: Animal Research: Reporting In Vivo Experiments
CONSORT: Consolidated Standards of Reporting Trials
FDA: Food and Drug Administration
ICMJE: International Committee of Medical Journal Editors
IQR: Interquartile range
ISRCTN: the International Standard Randomised Controlled Trial Number
OR: Odds ratio
PRISMA: Preferred Reporting Items for Systematic Reviews and Meta-Analyses
SPIRIT: Standard Protocol Items: Recommendations for Interventional Trials
STROBE: Strengthening the Reporting of Observational Studies in Epidemiology
WHO ICTRP: World Health Organization International Clinical Trials Registry Platform
